# Analysis of antihypertensive treatment using real-world Japanese data—the retrospective study of antihypertensives for lowering blood pressure (REAL) study

**DOI:** 10.1038/s41440-019-0238-2

**Published:** 2019-03-06

**Authors:** Mitsuru Ohishi, Takuo Yoshida, Akinori Oh, Shinzo Hiroi, Tomomi Takeshima, Yujiro Otsuka, Kosuke Iwasaki, Yukio Shimasaki

**Affiliations:** 1 Department of Cardiovascular Medicine and Hypertension, Graduate School of Medical and Dental Sciences, Kagoshima University, Kagoshima, Japan; 2 Japan Medical Affairs, Takeda Pharmaceutical Company Limited, Osaka, Japan; 3 Japan Medical Affairs, Takeda Pharmaceutical Company Limited, Tokyo, Japan; 4 Milliman Inc., Tokyo, Japan

**Keywords:** Angiotensin II receptor antagonists, Antihypertensive agents, Calcium channel blockers, Diuretics, Real-world data

## Abstract

Hypertension requires strict treatment because it causes diseases that can lead to death. Although various classes of antihypertensive drugs are available, the actual status of antihypertensive drug selection and the transition in prescription patterns over time have not been fully examined. Therefore, we conducted a claims-based study using two claims databases (2008–16) to determine this status in Japan. We examined the prescription rate for each class of antihypertensive drugs in hypertensive patients and compared the patients’ ages and the sizes of the medical institutions treating these patients. Among the 1 560 865 and 302 433 hypertensive patients in each database, calcium channel blockers (CCBs) (>60%) and angiotensin II receptor blockers (ARBs) (>55%) were the most frequently prescribed classes. The prescription rate of CCBs increased and ARBs decreased with the patients’ ages. Although the Japanese guidelines for management of hypertension in 2014 changed the recommendation and indicated that β-blockers should not be used as first-line drugs, their prescription status did not change during this study period up to 2016. Use of CCBs and ARBs as first-line drugs differed by the types of patient comorbidities. Although ARBs or angiotensin-converting enzyme inhibitors were recommended for patients with some comorbidities, CCBs were used relatively frequently. In conclusion, the patients’ ages and comorbidities and the sizes of the medical institutions affect the selection of antihypertensive drugs. Selection and use of drugs may not always follow the guidelines.

## Introduction

Sustained high blood pressure causes the development, progression, and recurrence of various cardiovascular diseases. High blood pressure also leads to a decrease in quality of life [1] and an increase in the risk of death [2, 3]. Antihypertensive treatments are administered to reduce such risks. Lifestyle modification is advised as a primary treatment. However, when the target blood pressure cannot be achieved by these modifications, antihypertensive drug therapy is required [4–6].

The Japanese Society of Hypertension (JSH) has published Guidelines for management of hypertension. The recommendation of first-choice antihypertensive drugs in these guidelines has changed over time. Diuretics were often used as a concomitant medicine in the JSH guidelines in 2004 [7] but were suggested as a first-choice drug together with other antihypertensive drug classes in the 2009 JSH guidelines [8]. β-blockers were recommended as one of the classes for first-line treatment in the 2009 JSH guidelines but were excluded from the first-line treatments in the 2014 JSH guidelines [9].

In the current Japanese guidelines, four classes of antihypertensive drugs [calcium channel blockers (CCBs), angiotensin II receptor blockers (ARBs), angiotensin-converting enzyme (ACE) inhibitors, and diuretics] are defined as first-choice drugs for hypertensive patients without compelling indications [9]. Antihypertensive treatment should be initiated with a single drug at a low dose, and a dose increase or combined use of more than one class is suggested when the first-line treatment is not sufficiently effective [9].

In a previous study, we examined the current status of antihypertensive treatments based on Japanese claims data [10]. In that study, we aimed to determine the prescription status of antihypertensive drugs of the ARB class prescribed as a first-line drug. We found that CCBs were most often prescribed as a second-line drug for patients receiving an ARB as a first-line drug [10]. The rate of prescribing a second-line drug differed by the type of ARB prescribed as the first-line drug. Additionally, diuretics were most often prescribed as a third-line drug for patients receiving combination therapy with an ARB and a CCB. The prescription rate for a third-line drug differed depending on the CCB dose prescribed as the second-line drug and the patient’s clinical history [10]. Another study reported that the prescription rate of diuretics did not change before and after 2009 based on a claims data analysis performed between 2005 and 2011 [11]. This outcome occurred even though the 2009 revised JSH guideline recommended the use of diuretics as a first-choice drug. The use of diuretics remains limited in patients taking multiple antihypertensive drugs based on data from a dispensing pharmacy in a local area in Japan [12]. This limited diuretics use occurred even though the guidelines recommended inclusion of diuretics, especially when three or more antihypertensive drugs were used.

As described above, real-world data analyses have been conducted to show the actual status of antihypertensive prescriptions. These analyses may allow examination of factors that affect the selection of antihypertensive drugs. However, comprehensive studies of treatment with antihypertensive drugs without limiting the prescription order and class have not been performed in Japan to date. Selection of antihypertensive drugs should be based on the patient’s background and clinical history. Therefore, understanding the current situation of antihypertensive drug prescription is essential and will allow appropriate treatment for each patient and effective use of health resources.

In the present study, we analyzed claims data to determine the comprehensive status of antihypertensive drug prescriptions in Japan. Specifically, we examined the prescription rate in each class, taking into account the patient backgrounds (age and clinical history), sizes of the medical institutions, and changes in the prescription rates by calendar year, to determine the factors that affected antihypertensive drug selection.

## Methods

### Study design and data sources

This study was a claims-based study that used two Japanese claims databases: Medical Data Vision (MDV; Tokyo, Japan) between April 2008 and April 2016 and Japan Medical Data Center (JMDC; Tokyo, Japan) between January 2010 and July 2016. We analyzed each database separately.

The MDV database consists of data from acute hospitals using the Japanese Diagnosis and Procedure Combination (DPC) fixed-payment reimbursement system and covers ~16 million individuals from 174 hospitals (11% of all DPC hospitals). Because the records do not contain unique, hospital-independent patient identifiers, we were unable to track treatments administered by other providers. The MDV database covers all patients receiving treatments in the hospital regardless of age and insurance type. Therefore, the MDV database was mainly used to compare treatment differences among patients categorized by age.

The JMDC database consists of data from individuals insured by health insurance societies and covers ~3.8 million individuals (5% of all insurers). Insured patients who are included in the JMDC database are company employees and their family members from more than 100 health insurance unions. Therefore, information for patients aged 65 years and older is limited, and no data are available for patients who are 75 years and older. Because the JMDC database included comprehensive records of all treatments, we were able to determine when antihypertensive drugs were not being prescribed to particular patients. Therefore, the first-line treatment could be identified in this database. The JMDC database was mainly used to compare differences among patients from medical institutions classified by size, including differences between clinics (defined as a setting with <20 beds) and hospitals (defined as a setting with ≥20 beds), and to examine the initially prescribed drugs and the second-line drugs added to the first-line drugs.

Excel 2010 (Microsoft, Redmond, WA, USA) and SAS version 9.4 (SAS Institute, Cary, NC, USA) were used for the statistical analyses.

This study was conducted in accordance with the Declaration of Helsinki. We used unlinkable anonymized data that were exempt from obtaining approval from the Institutional Review Board or Ethic Committee and individual informed consent. This study was conducted in accordance with the Ethical Guidelines for Medical and Health Research involving human subjects by the Ministry of Education, Culture, Sports, Science, and Technology and the Ministry of Health, Labour, and Welfare.

### Identification and analysis of patients

We identified patients who had at least one diagnosis of hypertension according to the International Classification of Diseases, 10th Revision (ICD-10, coded as I10–I15) [13]. Patients who received antihypertensive drugs at least once during the study period were included in this study. The classes of hypertensive drugs that were analyzed in this study were CCBs, ACE inhibitors, ARBs, diuretics, β-blockers, renin inhibitors, centrally acting sympatholytics, and vasodilators as defined by the Anatomical Therapeutic Chemical Classification System code. The prescription rate of each class was analyzed for each year between 2009 and 2015 for the MDV database and between 2010 and 2015 for the JMDC database. The prescription rate of the class (*k*) was calculated as follows:

Prescription rate of *k* = (∑_month in the year_ number of patients prescribed *k*)/(∑_month in the year_ number of patients prescribed any antihypertensives).

We defined the class of drugs initially prescribed as a single drug class following a 6-month period during which no antihypertensive drug prescription was recorded as a first-line class in the JMDC database. The prescription rate of each class as the first-line drug was calculated by dividing the number of patients who were initially prescribed a class of antihypertensive drugs, including a single class and a combination of two or more classes at once, by the number of patients who were initially prescribed any class of antihypertensive drugs.

For CCBs and ARBs, which were the most frequently prescribed first- and second-line antihypertensive drugs, the addition rate of a second- or third-line class was investigated among patients who were initially prescribed a standard dose of a single CCB or ARB. The most frequently prescribed dose of each compound was defined as the standard dose as follows: for CCBs, 5 mg for amlodipine, 8 mg for azelnidipine, 4 mg for benidipine, 10 mg for cilnidipine, 20 mg for nifedipine, and 100 mg for diltiazem; and for ARBs, 20 mg for azilsartan, 8 mg for candesartan, 100 mg for irbesartan, 50 mg for losartan, 20 mg for olmesartan, 40 mg for telmisartan, and 80 mg for valsartan.

The relationships between the initially prescribed antihypertensive drug class and comorbidities were examined using the JMDC database. Comorbidities were defined as diseases that were diagnosed during the 6 months before the initial antihypertensive prescription based on ICD-10 codes for the following conditions: cerebrovascular disease (CVD, I60–I69), angina (I20), heart failure (I50), myocardial infarction (I21–I22), chronic kidney disease (CKD, N18), fatty liver (K700 and K760), hyperlipidemia (E78), and type 2 diabetes mellitus (T2DM, E11–E14).

## Results

### Identification of patients

**Fig. 1:**
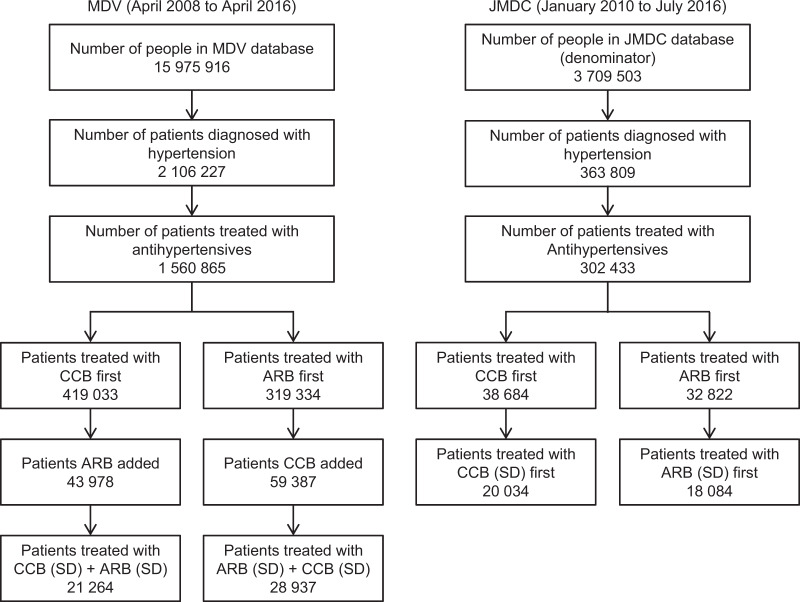
Identification of patients. MDV Medical Data Vision, JMDC Japan Medical Data Center, ARB angiotensin II receptor blocker, CCB calcium channel blocker, SD standard dose

**Fig. 2:**
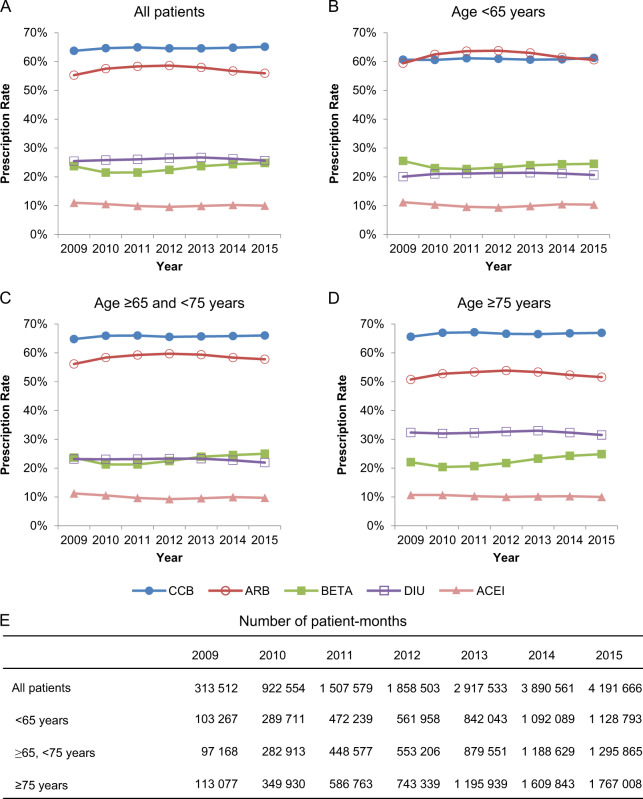
Prescription rates of antihypertensive drug classes in the MDV database. The five most frequently prescribed classes for **a** all patients and those in each age group, including patients aged **b** younger than 65 years, **c** 65 years to younger than 75 years, and **d** 75 years or older, and **e** the number of patient-months for **a**–**d**. MDV Medical Data Vision, ARB angiotensin II receptor blocker, ACEI angiotensin-converting enzyme inhibitor, BETA ß-blocker, CCB calcium channel blocker, DIU diuretic

**Fig. 3:**
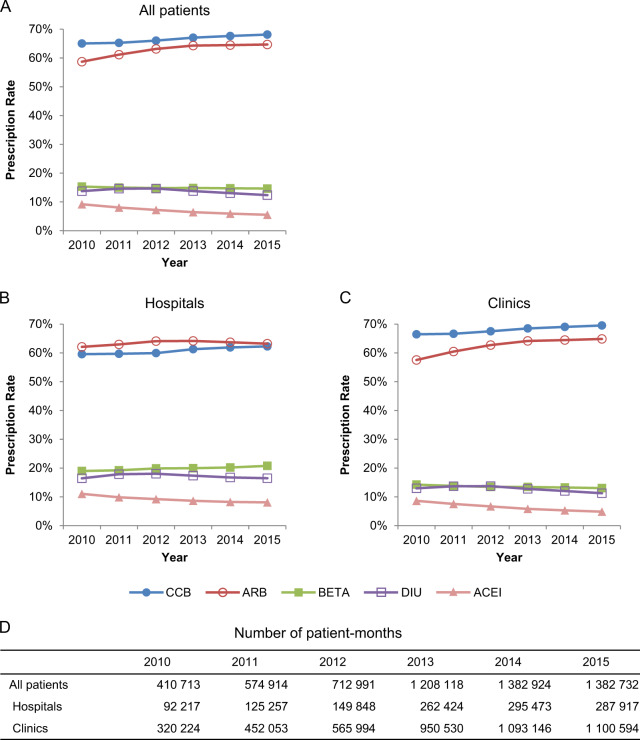
Prescription rates of antihypertensive drug classes in the JMDC database. The five most frequently prescribed classes for **a** all patient and those in **b** hospitals and **c** clinics, and **d** the number of patient-months for **a**–**c**. JMDC Japan Medical Data Center, ARB, angiotensin II receptor blocker, ACEI angiotensin-converting enzyme inhibitor, BETA ß-blocker, CCB calcium channel blocker, DIU diuretic

Approximately 2.10 and 0.36 million hypertensive patients were included in the MDV and JMDC databases, respectively. Among them were 1 560 865 unique patients in the MDV database and 302 433 unique patients in the JMDC database who were diagnosed with hypertension and treated with antihypertensive drugs over the entire data period (Fig. 1). The mean (standard deviation) age of all hypertensive patients in 2015 was 71.1 (13.2) years in the MDV database and 54.0 (10.6) years in the JMDC database (Supplementary Table 1). The male ratio was >50% for the whole study period in both databases (53.8% in the MDV database and 64.9% in the JMDC database in 2015) (Supplementary Table 1). The number of patient-months for each analysis of the prescription rate per year, which was the sum of patients in each month, is shown in Figs. 2e, 3d, and Supplementary Table 2. The numbers of unique patients who were prescribed a CCB or ARB at a standard dose as a first-line drug were 20 034 and 18 084, respectively, in the JMDC database. The numbers of patients who were prescribed a CCB followed by an ARB or an ARB followed by a CCB at a standard dose were 21 264 and 28 937, respectively, in the MDV database (Fig. 1). Prescription of antihypertensive drugs added as second-line drugs was examined in the JMDC database, and prescription of third-line drugs was examined in the MDV database.

### Prescription patterns of antihypertensive drugs

We investigated the prescription patterns of each antihypertensive drug class between 2009 and 2015 in the MDV database (Fig. 2a) and between 2010 and 2015 in the JMDC database (Fig. 3a). The number of patient-months in each year is shown in Fig. 2e (MDV database) and Fig. 3d (JMDC database). CCBs were the most frequently prescribed drug in the overall study period in the MDV and JMDC databases (63.7–5.1 and 65.0–8.1%, respectively), followed by ARBs (55.3–8.6 and 58.7–64.7%, respectively).

Comparison between age groups using the MDV database showed that the prescription rates of CCBs and ARBs were 60.5–1.3 and 59.4–63.8% for patients aged <65 years, 64.8–6.0 and 56.1–9.7% for those aged ≥65 and <75 years, and 65.6–7.1% and 50.8–3.9% for those aged ≥75 years, respectively (Fig. 2b–d). The CCB prescription rates gradually increased and the ARB prescription rates decreased with the increasing age of the patient. Diuretic drugs were more frequently prescribed in patients aged ≥75 years (31.5–3.0%) compared with those in the other age groups (20.1–1.4%, <65 years; 21.9–3.3%, ≥65 and <75 years) (Fig. 2b–d).

When we compared the differences in the sizes of the medical institutions in the JMDC database, we found that CCBs (66.5–9.5%) were the most frequently prescribed drug in clinics, followed by ARBs (57.5–64.9%). However, ARBs (62.1–4.2%) were prescribed more frequently than CCBs (59.6–62.3%) in hospitals (Fig. 3b, c). β-blockers and diuretics were more frequently prescribed in hospitals than in clinics (Fig. 3b, c).

### Prescription patterns of first-line antihypertensive classes

**Fig. 4:**
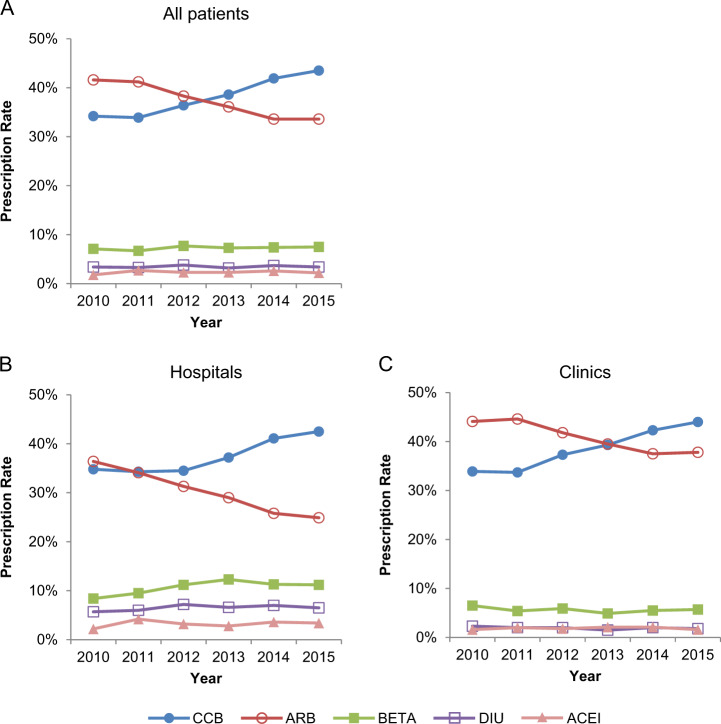
Prescription rates of first-line antihypertensive drug classes in the JMDC database. The five most frequently prescribed classes for **a** all patients and those in **b** hospitals and **c** clinics. JMDC Japan Medical Data Center, ARB angiotensin II receptor blocker, ACEI angiotensin-converting enzyme inhibitor, BETA ß-blocker, CCB calcium channel blocker, DIU diuretic

The number of patient-months for hypertensive patients who received first-line treatment with antihypertensive drugs as single or multiple classes were 2 325 in 2010 and 19 914 in 2015 in the JMDC database (Supplementary Table 2A). A change occurred in the rates of the initially prescribed antihypertensive class between 2010 and 2015. ARBs were the most frequently prescribed class in 2010, followed by CCBs. The prescription rate of ARBs as the first-line class decreased after 2011, with a concurrent increase in CCBs. Consequently, CCBs were more frequently prescribed than ARBs in and after 2013 (Fig. 4a). In 2015, CCBs were the most frequently prescribed drug (43.5%), followed by ARBs (33.6%), β-blockers (7.5%), diuretics (3.4%), and ACE inhibitors (2.2%) (Fig. 4a).

In the clinics, ARBs were more frequently prescribed as the first-line class than CCBs until 2012, were prescribed at the same rate as CCBs in 2013, and were exceeded by the prescription rate of CCBs starting in 2014. This trend resulted in a prescription rate of 44.0% for CCBs and 37.8% for ARBs in 2015 (Fig. 4c). In hospitals, the prescription rates of ARBs and CCBs were similar in 2011. However, the rate of CCBs increased and was higher than that of ARBs starting in 2012. The prescription rate was 42.5% for CCBs and 24.9% for ARBs in 2015 (Fig. 4b). β-blockers and diuretics were more frequently prescribed in hospitals than in clinics (Fig. 4b, c).

When the age groups (<65 years and ≥65 and <75 years) were compared in the JMDC database, the differences in first-line prescription rates were smaller than those between the sizes of the medical institutions. Some other differences were observed, such as the timing in which the prescription rate of CCBs exceeded that of ARBs (i.e., it was exceeded in 2013 for patients aged <65 years and in 2012 for those aged ≥65 and <75 years) (Supplementary Figure 1).

### Prescription patters for second- and third-line antihypertensive drugs

**Fig. 5:**
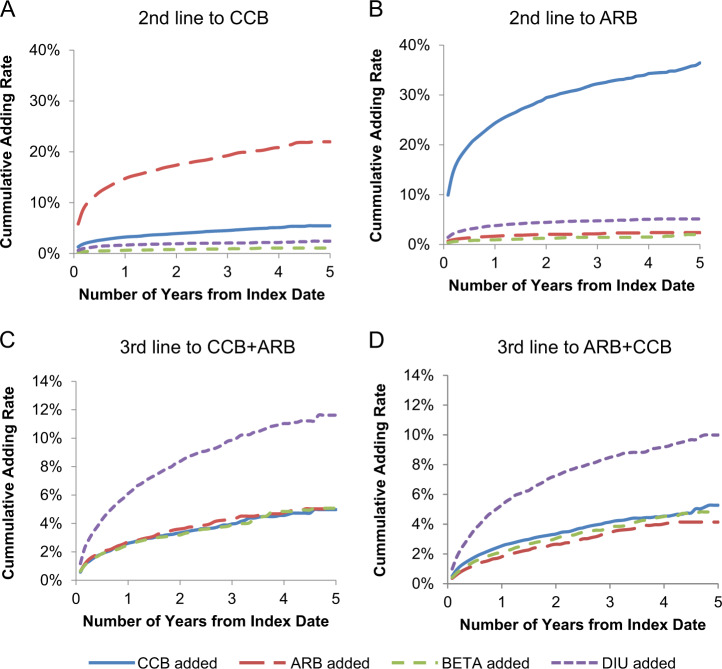
Addition rate of a second-line antihypertensive drug class to **a** CCBs or **b** ARBs in the JMDC database and a third-line antihypertensive to **c** CCB + ARB or **d** ARB + CCB in the MDV database. JMDC Japan Medical Data Center, MDV Medical Data Vision, ARB angiotensin II receptor blocker, BETA ß-blocker, CCB calcium channel blocker, DIU diuretic

For CCBs and ARBs that were initially prescribed as a standard dose, the most frequently prescribed second-line drug classes were ARBs and CCBs, respectively, according to the JMDC database (Fig. 5a, b). The addition rate of CCBs as second-line drugs to patients initially prescribed ARBs was greater than 20% within 1 year and 32.2% within 3 years (Fig. 5b). However, the addition rate of ARBs as second-line drugs to CCBs was less than 20% within 3 years (Fig. 5a).

For the combinations of an ARB as the first-line drug and a CCB as the second-line drug (ARB + CCB) and a CCB as the first-line drug and an ARB as the second-line drug (CCB + ARB), the most frequently used third-line drug was a diuretic among all of the classes in the MDV database (Fig. 5c, d). The addition rate of diuretics reached ~10% within 3 years for CCB + ARB and within 5 years for ARB + CCB (Fig. 5c, d).

### Relationships between the initial antihypertensive class and comorbidities

**Fig. 6:**
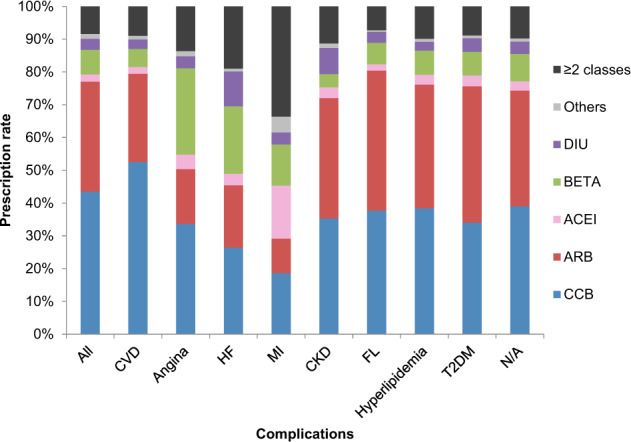
Composition of the initial antihypertensive drug classes by comorbid conditions in the JMDC database (2015). JMDC Japan Medical Data Center, CKD chronic kidney disease, CVD cerebrovascular disease, FL fatty liver, HF heart failure, MI myocardial infarction, N/A not available, T2DM type 2 diabetes mellitus, ARB angiotensin II receptor blocker, ACEI angiotensin-converting enzyme inhibitor, BETA ß-blocker, CCB calcium channel blocker, DIU diuretic

The composition of classes that were initially prescribed antihypertensive drugs differed depending on patient comorbidities in the JMDC database (Fig. 6). CCBs were more frequently prescribed than ARBs for patients with CVD, angina, heart failure, and myocardial infarction. CCBs were prescribed at a level similar to ARBs for patients with CKD and hyperlipidemia. ARBs were more frequently prescribed for patients with a fatty liver and T2DM. β-blockers were the second most frequently prescribed class following CCBs for patients with angina and heart failure.

For a more detailed analysis, we focused on patients with CKD, because the inconsistency with the recommendations in the guidelines was remarkable. We analyzed the prescription rate of first-line antihypertensive drugs in patients with CKD in each year. The prescription rate of ARBs as the first-line drug class was higher than that of CCBs, and the prescription rates moved closer together over time. The prescription rate of ARBs (47% in 2010 and 34% in 2014) was ~5–10% higher than that of CCBs by 2014 and was almost the same as that of CCBs in 2015 in the JMDC database (Supplementary Figure 2). First-line prescription of diuretics ranged from 8–14% in the most recent 4 years (Supplementary Figure 2).

Regarding the prescription of drugs for hypertensive patients with CKD regardless of the first-line drugs, diuretics were frequently prescribed (>40%) following CCBs and ARBs in the MDV database (Supplementary Figure 3A). The frequency of prescription for diuretics and β-blockers was similar (>25%) in the JMDC database (Supplementary Figure 3B). For the former, the patterns in the MDV database were different from those for all hypertensive patients (Fig. 2a). We also observed a difference in the prescription of drugs with the size of the medical institution. Diuretics were more frequently prescribed in hospitals than in clinics in the JMDC database (Supplementary Figure 3C, S3D).

## Discussion

In this study, we examined the overall status of treatment with antihypertensive drugs in Japan based on a real-world data analysis. CCBs and ARBs were the most frequently prescribed drugs regardless of the initial treatment during the entire study period. This finding may be due to their reliability, efficacy in reducing blood pressure and safety profiles. Furthermore, the combination of these classes represented the majority of prescriptions of first- and second-line antihypertensive drugs. In a previous study, we reported several possible reasons for these findings as follows: (1) the availability of many fixed-dose combinations of ARBs and CCBs, (2) the popularity of each CCB and ARB as antihypertensive drugs, and (3) combination therapy with ARBs and CCBs has become the most popular regimen for antihypertensive treatment in Japan, because its enhanced blood pressure-lowering effect reduces the risk of cardiovascular events [10]. Regarding the trends in initially prescribed antihypertensive drugs, an increase in CCBs and a decrease in ARBs were observed from 2011 onwards. This decrease in the prescription of ARBs may have been caused by retraction of some clinical studies of ARBs that were conducted in Japan [14].

Although diuretics are recommended as major antihypertensive drugs, the prescription rate in the current study was relatively low compared with those of ARBs and CCBs. The achievement rate of target blood pressure is still low in Japan [9, 15], which may be related to the lower prescription rate of diuretics observed in this study. The low preference for diuretics is probably due to their side effects, including impaired glucose tolerance and hypokalemia. However, diuretics have been reported as a necessary drug for intensive hypertensive treatment. In the SPRINT trial, diuretics were more frequently prescribed for the intensive treatment group (67.0%) than for the standard treatment group (42.9%) [16]. The prescription led to achievement of a lower blood pressure goal (<120 mm Hg systolic blood pressure), which in turn resulted in lower rates of major cardiovascular events and death. A randomized controlled trial showed that adding diuretics to a single pill fixed-dose combination of an ARB and CCB was effective, similar to an increase in the CCB dose [17]. A subanalysis of a clinical trial performed in Japan suggested that the combination of an ARB with a diuretic might provide better cardiovascular outcomes than combination with a β-blocker, even in patients with poor blood pressure control [18]. The 2009 JSH guidelines recommended using diuretics as a first-line antihypertensive drug [8]. However, as reported by Kohro et al., prescription of diuretics did not increase after the change in the guidelines [11]. In this study, we showed that the prescription rate of diuretics did not change from 2010 onward. An observational study conducted in a local district in Japan in 2014 also reported that prescription of diuretics was still low, even for patients who were prescribed multiple antihypertensive drugs [12]. This trend occurred even though the JSH guidelines recommended including diuretics when multiple classes of antihypertensive drugs were used [9]. Considering the current low achievement rate of the blood pressure goal in Japan, the use of multiple classes of antihypertensive drugs, including diuretics as mentioned in the guidelines [9], should be considered.

Conversely, β-blockers were still frequently prescribed together with CCBs and ARBs in 2015 as a first-line choice in this study. This finding conflicts with the change in the recommendation suggested in the JSH guidelines in 2014, which did not recommend the use of β-blockers for initial treatment unless the patient had a compelling indication for their use. These observations suggest that the treatment guidelines may not always affect the selection and use of antihypertensive drugs. This real-world data analysis enables us to unveil the actual treatment status, which may be useful for reconsidering treatment based on the recommendation in the guidelines.

In our study, prescription patterns for antihypertensive drugs differed depending on the patients’ ages and comorbidities and the sizes of the medical institutions. Prescription rates of CCBs consistently increased with age, and diuretics were more frequently prescribed for patients aged ≥75 years. The rise in prescription of CCBs with age might be related to the effectiveness of CCBs for patients with decreased vascular elasticity [19]. In the National Institute for Health and Care Excellence guidelines, CCBs are recommended as the first-line medication for patients aged ≥55 years [20], although CCBs, ARBs, ACE inhibitors, and thiazide diuretics are recommended for older hypertensive patients in the guidelines published by the Japan Geriatrics Society in 2017 [21]. Additionally, an increase in diuretic use with age may be related to preventing fractures according to the JSH guideline in 2014 [9] and to higher salt sensitivity in older patients [22, 23].

Use of CCBs and ARBs as first-line antihypertensive drugs differed by the types of comorbidities in our study. According to the 2014 JSH guidelines, ARBs or ACE inhibitors are recommended for patients with heart failure, whereas CCBs require careful use [9]. Only ARBs or ACE inhibitors are recommended for first-line treatment in patients with diabetes mellitus and CKD (with proteinuria), whereas CCBs are not recommended [9]. Nevertheless, in our study, CCBs were the most frequently prescribed antihypertensive drug in patients with heart failure and were often prescribed for those with CKD and T2DM. We analyzed the prescription patterns for hypertensive patients with CKD in detail. We found that the prescription rate of ARBs was the same or lower than that of CCBs, even for patients with CKD, regardless of initial treatment and size of the medical institution. This finding suggests that antihypertensive drugs may be selected without consulting the JSH guidelines.

CCBs were more frequently prescribed in clinics than in hospitals in our study. ARBs were prescribed at the same rate as CCBs in hospitals, and the other classes were more frequently prescribed in hospitals than in clinics. These differences are probably because hospitals have more patients with comorbidities and more specialists. Therefore, treatments considering comorbidities are more likely in hospitals than in clinics.

This study has several limitations, because these two databases have potential biases. The JMDC database only included information for company employees and their family members. Therefore, the age distribution in this database differs from that of the general patient population. Additionally, data from patients who were self-employed and employees in small- and medium-sized enterprises were not included. The MDV database only includes data from DPC hospitals. Therefore, patients with a severe condition and those with comorbidities may be overrepresented compared with those in the general patient population. Moreover, treatments in other settings and data after changes in settings could not be followed in the MDV database. However, although the patient composition was different between the two databases, the prescription patterns of antihypertensive drug classes in all patients were similar (Supplementary Figure 2A and 3A). Because the patients’ chart data were not available in either database, the accuracy of their diagnoses based on ICD-10 codes could not be evaluated. Furthermore, although we examined the prescription of antihypertensive drugs for hypertensive patients, the prescriptions may not have been prescribed for treatment for hypertension, because some drugs have indications for treatment for other diseases. Additionally, the study period up to 2016 may have been too short to observe the effect of a change in the guidelines in 2014 on the actual selection of drugs. A change in prescriptions due to a change in the guidelines may be seen several years later.

In summary, this study comprehensively analyzed the prescription status of antihypertensive drugs in Japanese hypertensive patients. We found that prescription choices often differed from the guidelines for managing hypertension. Consequently, this real-world data analysis may be an indicator of the degree of infiltration of the guidelines into actual clinical practice. Detailed analyses including the patient’s age and the size of the medical institutions as well as the types of comorbidities can provide important information for considering the selection of appropriate treatment for each patient. We believe that additional analyses, particularly with a focus on comorbidities of hypertensive patients, are required in the future.

## Supplementary information

Supplementary material
