# Supplementary material for: Analysis of antihypertensive treatment using real-world Japanese data—the retrospective study of antihypertensives for lowering blood pressure (REAL) study
Source: Hypertens Res. 2019 Mar 6;42(7):1057–67. doi: 10.1038/s41440-019-0238-2 (PMC8075880; doi:10.1038/s41440-019-0238-2)
Supplement: Supplementary file 1 — Supplementary material [file 41440_2019_238_MOESM1_ESM.pptx]

## Slide 1
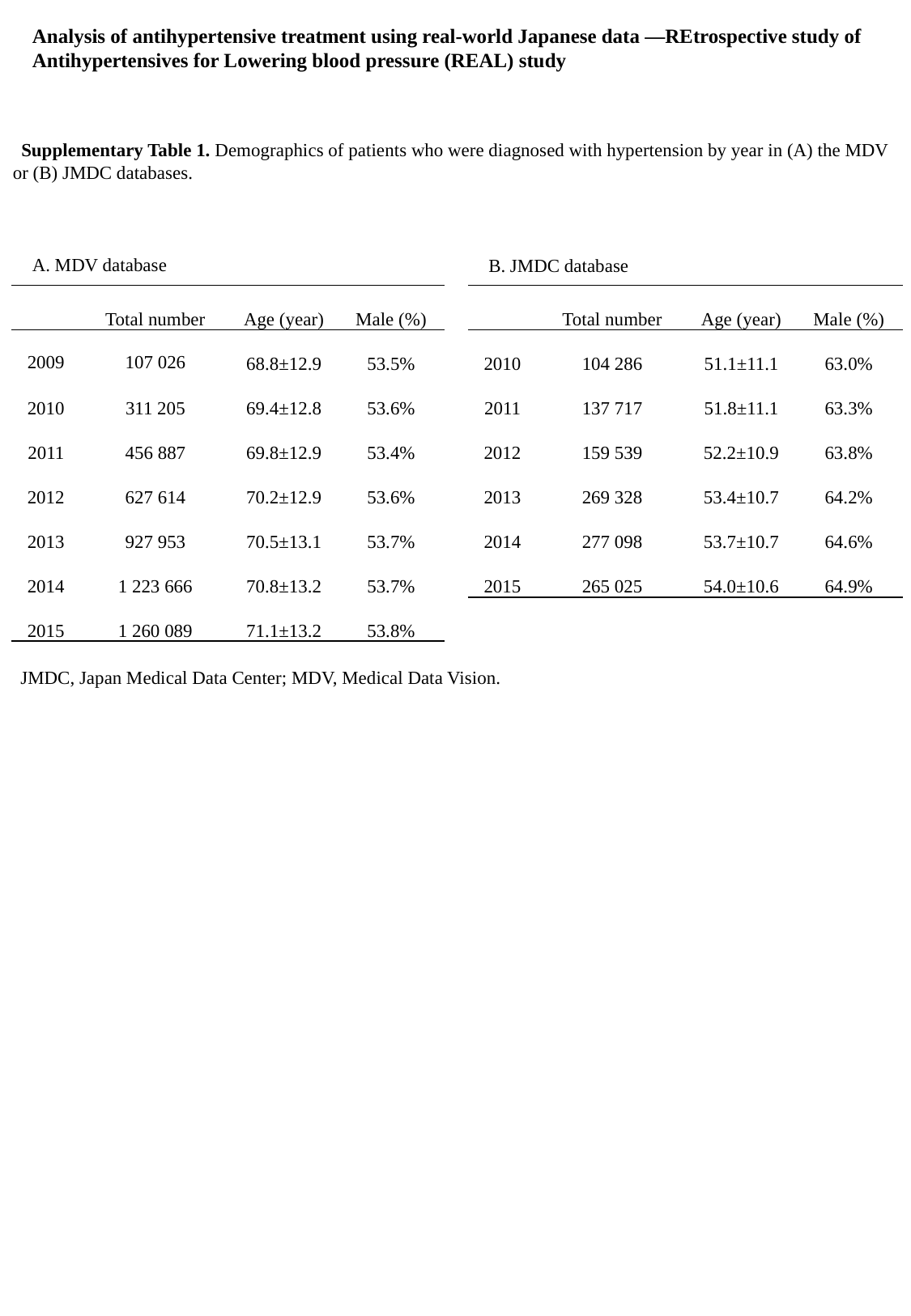

Analysis of antihypertensive treatment using real-world Japanese data ―REtrospective study of Antihypertensives for Lowering blood pressure (REAL) study
Supplementary Table 1. Demographics of patients who were diagnosed with hypertension by year in (A) the MDV or (B) JMDC databases.
A. MDV database
B. JMDC database
| | Total number | Age (year) | Male (%) |
| --- | --- | --- | --- |
| 2009 | 107 026 | 68.8±12.9 | 53.5% |
| 2010 | 311 205 | 69.4±12.8 | 53.6% |
| 2011 | 456 887 | 69.8±12.9 | 53.4% |
| 2012 | 627 614 | 70.2±12.9 | 53.6% |
| 2013 | 927 953 | 70.5±13.1 | 53.7% |
| 2014 | 1 223 666 | 70.8±13.2 | 53.7% |
| 2015 | 1 260 089 | 71.1±13.2 | 53.8% |
| | Total number | Age (year) | Male (%) |
| --- | --- | --- | --- |
| 2010 | 104 286 | 51.1±11.1 | 63.0% |
| 2011 | 137 717 | 51.8±11.1 | 63.3% |
| 2012 | 159 539 | 52.2±10.9 | 63.8% |
| 2013 | 269 328 | 53.4±10.7 | 64.2% |
| 2014 | 277 098 | 53.7±10.7 | 64.6% |
| 2015 | 265 025 | 54.0±10.6 | 64.9% |
JMDC, Japan Medical Data Center; MDV, Medical Data Vision.

## Slide 2
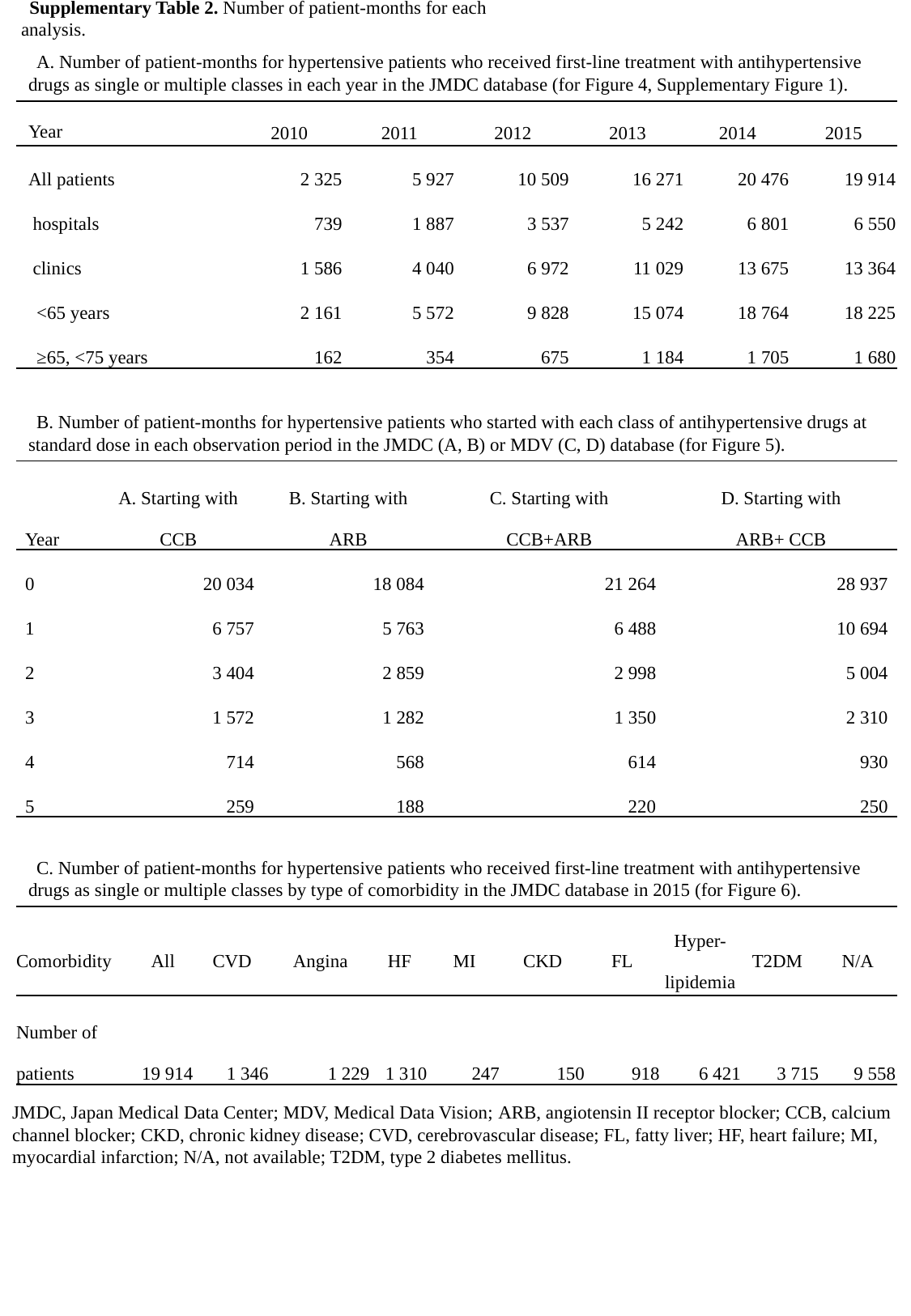

Supplementary Table 2. Number of patient-months for each analysis.
A. Number of patient-months for hypertensive patients who received first-line treatment with antihypertensive drugs as single or multiple classes in each year in the JMDC database (for Figure 4, Supplementary Figure 1).
| Year | 2010 | 2011 | 2012 | 2013 | 2014 | 2015 |
| --- | --- | --- | --- | --- | --- | --- |
| All patients | 2 325 | 5 927 | 10 509 | 16 271 | 20 476 | 19 914 |
| hospitals | 739 | 1 887 | 3 537 | 5 242 | 6 801 | 6 550 |
| clinics | 1 586 | 4 040 | 6 972 | 11 029 | 13 675 | 13 364 |
| <65 years | 2 161 | 5 572 | 9 828 | 15 074 | 18 764 | 18 225 |
| ≥65, <75 years | 162 | 354 | 675 | 1 184 | 1 705 | 1 680 |
B. Number of patient-months for hypertensive patients who started with each class of antihypertensive drugs at standard dose in each observation period in the JMDC (A, B) or MDV (C, D) database (for Figure 5).
| Year | A. Starting with CCB | B. Starting with ARB | C. Starting with CCB+ARB | D. Starting with ARB+ CCB |
| --- | --- | --- | --- | --- |
| 0 | 20 034 | 18 084 | 21 264 | 28 937 |
| 1 | 6 757 | 5 763 | 6 488 | 10 694 |
| 2 | 3 404 | 2 859 | 2 998 | 5 004 |
| 3 | 1 572 | 1 282 | 1 350 | 2 310 |
| 4 | 714 | 568 | 614 | 930 |
| 5 | 259 | 188 | 220 | 250 |
C. Number of patient-months for hypertensive patients who received first-line treatment with antihypertensive drugs as single or multiple classes by type of comorbidity in the JMDC database in 2015 (for Figure 6).
| Comorbidity | All | CVD | Angina | HF | MI | CKD | FL | Hyper- lipidemia | T2DM | N/A |
| --- | --- | --- | --- | --- | --- | --- | --- | --- | --- | --- |
| Number of patients | 19 914 | 1 346 | 1 229 | 1 310 | 247 | 150 | 918 | 6 421 | 3 715 | 9 558 |
JMDC, Japan Medical Data Center; MDV, Medical Data Vision; ARB, angiotensin II receptor blocker; CCB, calcium channel blocker; CKD, chronic kidney disease; CVD, cerebrovascular disease; FL, fatty liver; HF, heart failure; MI, myocardial infarction; N/A, not available; T2DM, type 2 diabetes mellitus.

## Slide 3
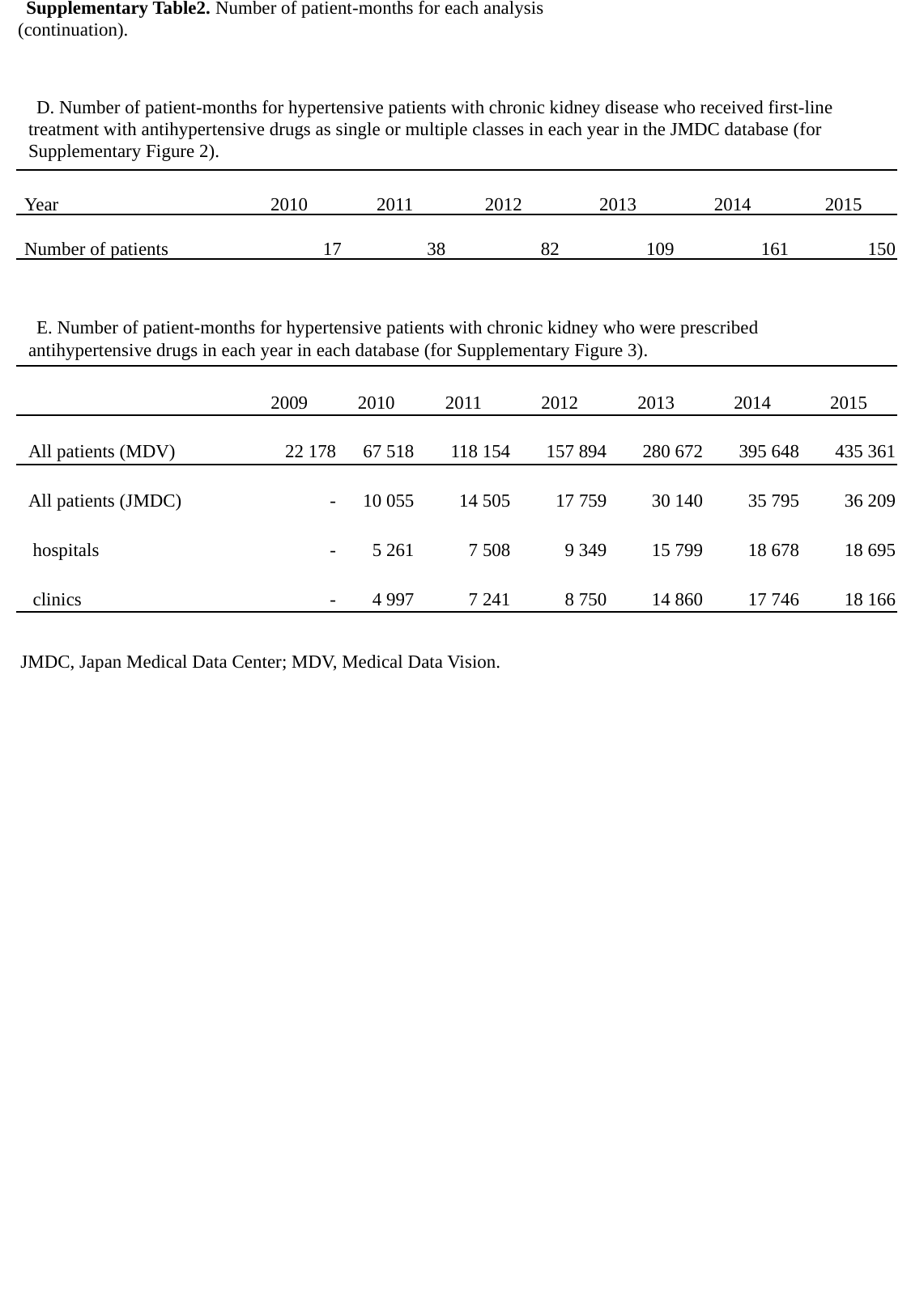

Supplementary Table2. Number of patient-months for each analysis (continuation).
D. Number of patient-months for hypertensive patients with chronic kidney disease who received first-line treatment with antihypertensive drugs as single or multiple classes in each year in the JMDC database (for Supplementary Figure 2).
| Year | 2010 | 2011 | 2012 | 2013 | 2014 | 2015 |
| --- | --- | --- | --- | --- | --- | --- |
| Number of patients | 17 | 38 | 82 | 109 | 161 | 150 |
E. Number of patient-months for hypertensive patients with chronic kidney who were prescribed antihypertensive drugs in each year in each database (for Supplementary Figure 3).
| | 2009 | 2010 | 2011 | 2012 | 2013 | 2014 | 2015 |
| --- | --- | --- | --- | --- | --- | --- | --- |
| All patients (MDV) | 22 178 | 67 518 | 118 154 | 157 894 | 280 672 | 395 648 | 435 361 |
| All patients (JMDC) | - | 10 055 | 14 505 | 17 759 | 30 140 | 35 795 | 36 209 |
| hospitals | - | 5 261 | 7 508 | 9 349 | 15 799 | 18 678 | 18 695 |
| clinics | - | 4 997 | 7 241 | 8 750 | 14 860 | 17 746 | 18 166 |
JMDC, Japan Medical Data Center; MDV, Medical Data Vision.

## Slide 4
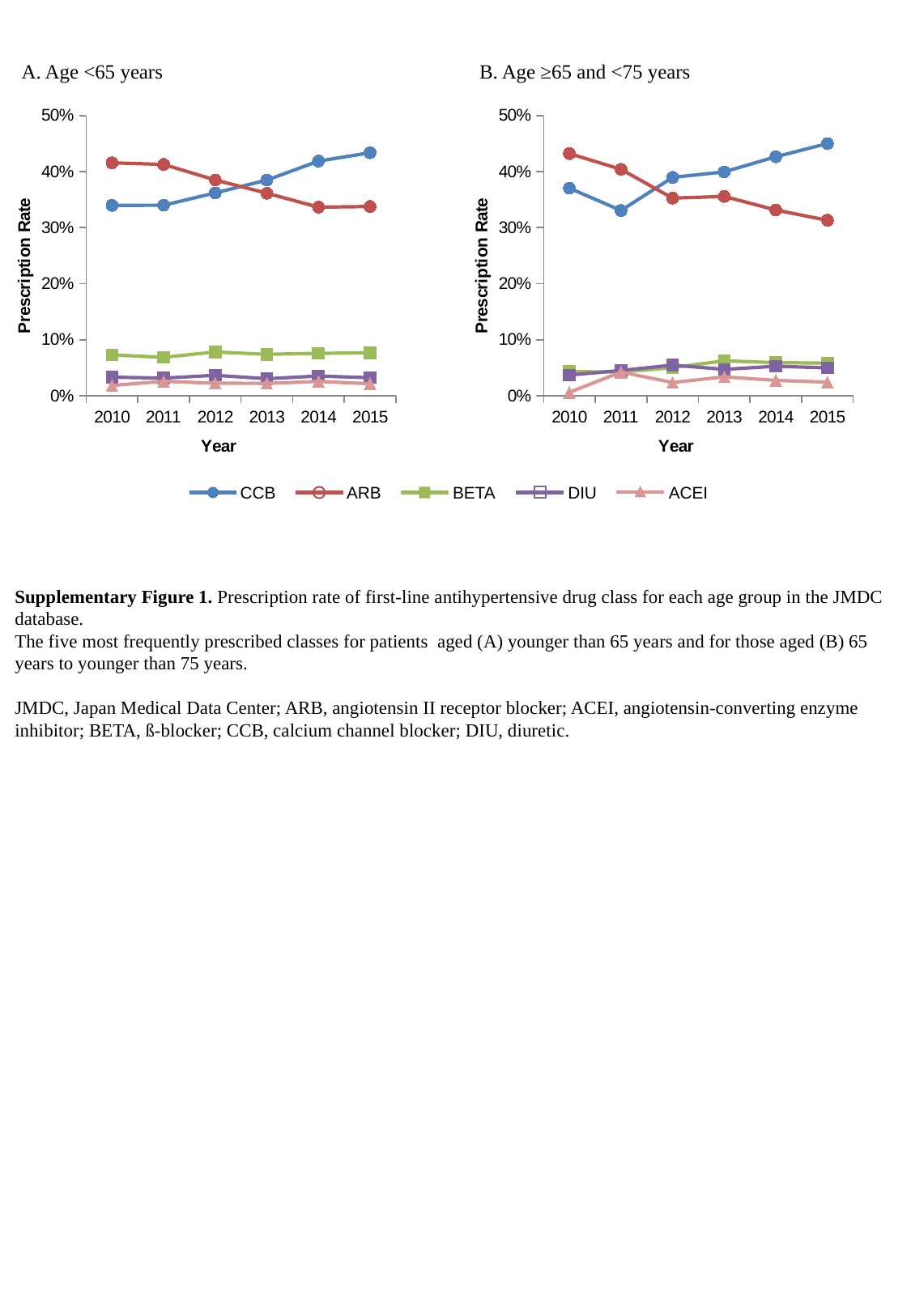

A. Age <65 years
B. Age ≥65 and <75 years
### Chart
| Category | CCB | ARB | BETA | DIU | ACE |
|---|---|---|---|---|---|
| 2010 | 0.33965756594169366 | 0.4155483572420176 | 0.07311429893567793 | 0.033317908375751965 | 0.01850994909763998 |
| 2011 | 0.3399138549892319 | 0.41277817659727206 | 0.06873653984206748 | 0.03158650394831299 | 0.025843503230437905 |
| 2012 | 0.3619251119251119 | 0.385022385022385 | 0.07824582824582825 | 0.03663003663003663 | 0.022486772486772486 |
| 2013 | 0.3848348149130954 | 0.3612843306355314 | 0.0741011012339127 | 0.030516120472336473 | 0.022223696430940694 |
| 2014 | 0.41867405670432745 | 0.3364954167554892 | 0.07589000213174163 | 0.035333617565551055 | 0.02558089959496909 |
| 2015 | 0.43358024691358027 | 0.3376131687242798 | 0.07670781893004115 | 0.032263374485596706 | 0.021618655692729766 |
### Chart
| Category | CCB | ARB | BETA | DIU | ACE |
|---|---|---|---|---|---|
| 2010 | 0.37037037037037035 | 0.43209876543209874 | 0.043209876543209874 | 0.037037037037037035 | 0.006172839506172839 |
| 2011 | 0.3305084745762712 | 0.403954802259887 | 0.0423728813559322 | 0.04519774011299435 | 0.0423728813559322 |
| 2012 | 0.3896296296296296 | 0.35259259259259257 | 0.05037037037037037 | 0.054814814814814816 | 0.023703703703703703 |
| 2013 | 0.39949324324324326 | 0.35557432432432434 | 0.0625 | 0.0472972972972973 | 0.033783783783783786 |
| 2014 | 0.42639296187683284 | 0.3313782991202346 | 0.05923753665689149 | 0.05278592375366569 | 0.02756598240469208 |
| 2015 | 0.45 | 0.3130952380952381 | 0.058333333333333334 | 0.05 | 0.024404761904761905 |CCB
ARB
BETA
DIU
ACEI
Supplementary Figure 1. Prescription rate of first-line antihypertensive drug class for each age group in the JMDC database.
The five most frequently prescribed classes for patients aged (A) younger than 65 years and for those aged (B) 65 years to younger than 75 years.
JMDC, Japan Medical Data Center; ARB, angiotensin II receptor blocker; ACEI, angiotensin-converting enzyme inhibitor; BETA, ß-blocker; CCB, calcium channel blocker; DIU, diuretic.

## Slide 5
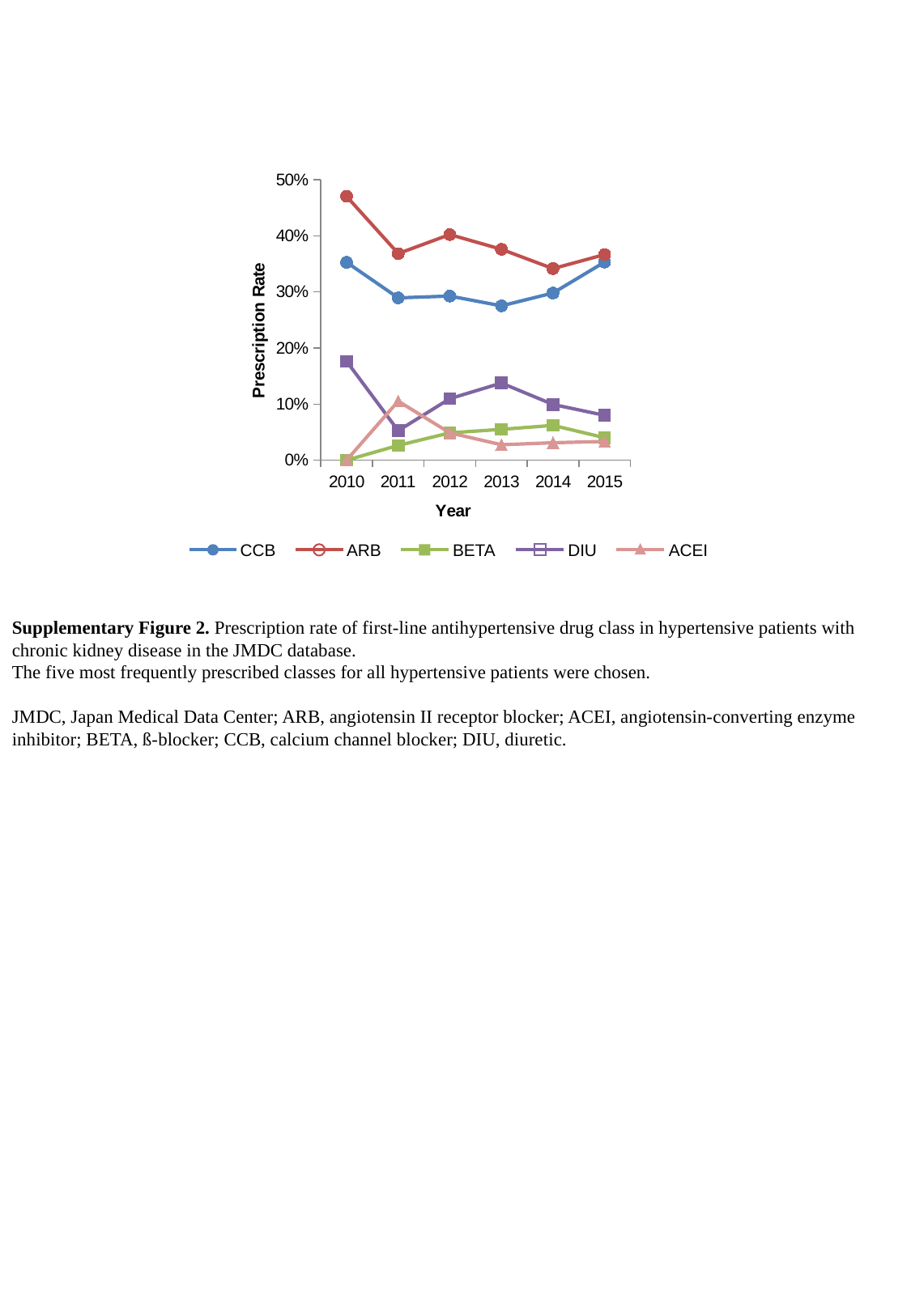

### Chart
| Category | CCB | ARB | BETA | DIU | ACE |
|---|---|---|---|---|---|
| 2010 | 0.35294117647058826 | 0.47058823529411764 | 0.0 | 0.17647058823529413 | 0.0 |
| 2011 | 0.2894736842105263 | 0.3684210526315789 | 0.02631578947368421 | 0.05263157894736842 | 0.10526315789473684 |
| 2012 | 0.2926829268292683 | 0.4024390243902439 | 0.04878048780487805 | 0.10975609756097561 | 0.04878048780487805 |
| 2013 | 0.27522935779816515 | 0.3761467889908257 | 0.05504587155963303 | 0.13761467889908258 | 0.027522935779816515 |
| 2014 | 0.2981366459627329 | 0.3416149068322981 | 0.062111801242236024 | 0.09937888198757763 | 0.031055900621118012 |
| 2015 | 0.35333333333333333 | 0.36666666666666664 | 0.04 | 0.08 | 0.03333333333333333 |CCB
ARB
BETA
DIU
ACEI
Supplementary Figure 2. Prescription rate of first-line antihypertensive drug class in hypertensive patients with chronic kidney disease in the JMDC database.
The five most frequently prescribed classes for all hypertensive patients were chosen.
JMDC, Japan Medical Data Center; ARB, angiotensin II receptor blocker; ACEI, angiotensin-converting enzyme inhibitor; BETA, ß-blocker; CCB, calcium channel blocker; DIU, diuretic.

## Slide 6
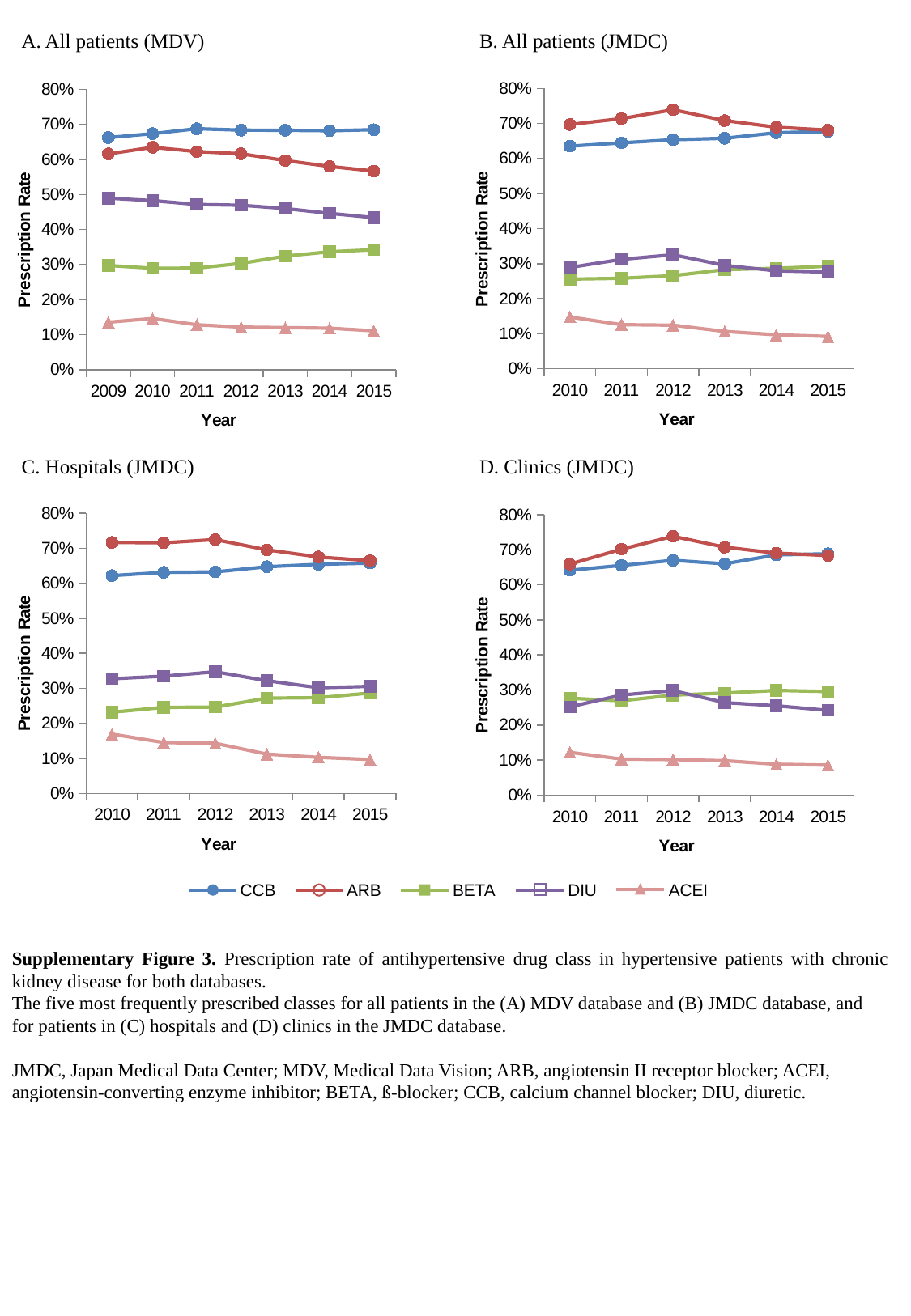

A. All patients (MDV)
B. All patients (JMDC)
### Chart
| Category | CCB | ARB | BETA | DIU | ACE |
|---|---|---|---|---|---|
| 2010 | 0.6353058180009945 | 0.6971655892590751 | 0.25589259075087023 | 0.28910989557434114 | 0.1477871705619095 |
| 2011 | 0.6448121337469838 | 0.713891761461565 | 0.2581868321268528 | 0.31216821785591176 | 0.12588762495691141 |
| 2012 | 0.6538093361112676 | 0.7391181935919815 | 0.26566811194324 | 0.3254124669181823 | 0.12438763443887606 |
| 2013 | 0.6577969475779695 | 0.7082946250829463 | 0.28281353682813537 | 0.29499004644990046 | 0.10650298606502986 |
| 2014 | 0.6735018857382316 | 0.6893141500209526 | 0.2867718955161335 | 0.27964799553010194 | 0.0969688503981003 |
| 2015 | 0.6771520892595764 | 0.681073766190726 | 0.29260681046148745 | 0.27598110966886685 | 0.09235272998425806 |
### Chart
| Category | CCB | ARB | BETA | DIU | ACE |
|---|---|---|---|---|---|
| 2009 | 0.6625935611867616 | 0.616015871584453 | 0.29750202903778517 | 0.48962936243123817 | 0.13581026242222022 |
| 2010 | 0.6736573950650196 | 0.6348233063775586 | 0.28962646997837616 | 0.48256761159986966 | 0.14621286175538376 |
| 2011 | 0.6877803544526635 | 0.6226027049443946 | 0.2899097787633089 | 0.4716133182118252 | 0.1284425410904413 |
| 2012 | 0.683743524136446 | 0.6163882098116458 | 0.303501083005054 | 0.4693845237944444 | 0.12164490100953805 |
| 2013 | 0.6833243073765819 | 0.5970171588188348 | 0.32416129859765136 | 0.4599318777790446 | 0.12012954623190059 |
| 2014 | 0.6821265367195083 | 0.5801748018440634 | 0.33657442979618246 | 0.4462325097055969 | 0.11851443707538013 |
| 2015 | 0.6850360964808515 | 0.566943295334217 | 0.342444546020429 | 0.4340237182476152 | 0.11104347886007244 |C. Hospitals (JMDC)
D. Clinics (JMDC)
### Chart
| Category | CCB | ARB | BETA | DIU | ACE |
|---|---|---|---|---|---|
| 2010 | 0.6217449154153203 | 0.7164037255274662 | 0.23189507698156245 | 0.3273141988215168 | 0.1693594373693214 |
| 2011 | 0.630927011188066 | 0.7153702717101759 | 0.2456046883324454 | 0.33470964304741607 | 0.14544485881726157 |
| 2012 | 0.6322601347737726 | 0.7244625093592898 | 0.24665739651299604 | 0.3472029094020751 | 0.14322387421114557 |
| 2013 | 0.6471295651623521 | 0.695170580416482 | 0.2720425343376163 | 0.32172922336856763 | 0.11234888284068612 |
| 2014 | 0.6539244030410108 | 0.6748581218545883 | 0.2733162008780383 | 0.30174536888317804 | 0.1031695042295749 |
| 2015 | 0.6574485156458946 | 0.6641882856378711 | 0.2864937148970313 | 0.30548274939823483 | 0.09697780155121691 |
### Chart
| Category | CCB | ARB | BETA | DIU | ACE |
|---|---|---|---|---|---|
| 2010 | 0.6417850710426256 | 0.6591955173103863 | 0.27656593956373826 | 0.25195117070242146 | 0.12207324394636782 |
| 2011 | 0.6557105372186162 | 0.7018367628780555 | 0.26929982046678635 | 0.2858721171108963 | 0.10302444413755006 |
| 2012 | 0.6701714285714285 | 0.7386285714285714 | 0.2854857142857143 | 0.29828571428571427 | 0.10148571428571429 |
| 2013 | 0.660228802153432 | 0.7077388963660834 | 0.29131897711978466 | 0.26406460296096906 | 0.09838492597577389 |
| 2014 | 0.6857319959427477 | 0.6905218077313198 | 0.29877155415304857 | 0.25509974078665615 | 0.0882452383635749 |
| 2015 | 0.6888693163051856 | 0.6838049102719366 | 0.295662226136739 | 0.2419905317626335 | 0.08565451943190576 |CCB
ARB
BETA
DIU
ACEI
Supplementary Figure 3. Prescription rate of antihypertensive drug class in hypertensive patients with chronic kidney disease for both databases.
The five most frequently prescribed classes for all patients in the (A) MDV database and (B) JMDC database, and for patients in (C) hospitals and (D) clinics in the JMDC database.
JMDC, Japan Medical Data Center; MDV, Medical Data Vision; ARB, angiotensin II receptor blocker; ACEI, angiotensin-converting enzyme inhibitor; BETA, ß-blocker; CCB, calcium channel blocker; DIU, diuretic.
